# Polyphenol-Rich Fraction of Brown Alga *Ecklonia cava* Collected from Gijang, Korea, Reduces Obesity and Glucose Levels in High-Fat Diet-Induced Obese Mice

**DOI:** 10.1155/2012/418912

**Published:** 2012-07-12

**Authors:** Eun Young Park, Eung Hwi Kim, Mi Hwi Kim, Young Wan Seo, Jung Im Lee, Hee Sook Jun

**Affiliations:** ^1^College of Pharmacy, Gachon University, Incheon 406-840, Republic of Korea; ^2^Lee Gil Ya Cancer and Diabetes Institute, Gachon University, Incheon 406-840, Republic of Korea; ^3^Division of Marine Environment and Bioscience, Korea Maritime University, Busan 606-791, Republic of Korea

## Abstract

*Ecklonia cava (E. cava)* is a brown alga that has beneficial effects in models of type 1 and type 2 diabetes. However, the effects of *E. cava* extracts on diet-induced obesity and type 2 diabetes have not been specifically examined. We investigated the effects of *E. cava* on body weight, fat content, and hyperglycemia in high-fat diet- (HFD) induced obese mice and sought the mechanisms involved. C57BL/6 male mice were fed a HFD (60% fat) diet or normal chow. After 3 weeks, the HFD diet group was given extracts (200 mg/kg) of *E. cava* harvested from Jeju (CA) or Gijang (G-CA), Korea or PBS by oral intubation for 8 weeks. Body weights were measured weekly. Blood glucose and glucose tolerance were measured at 7 weeks, and fat pad content and mRNA expression of adipogenic genes and inflammatory cytokines were measured after 8 weeks of treatment. G-CA was effective in reducing body weight gain, body fat, and hyperglycemia and improving glucose tolerance as compared with PBS-HFD mice. The mRNA expression of adipogenic genes was increased, and mRNA expression of inflammatory cytokines and macrophage marker gene was decreased in G-CA-treated obese mice. We suggest that G-CA reduces obesity and glucose levels by anti-inflammatory actions and improvement of lipid metabolism.

## 1. Introduction

The incidence of obesity has increased at an epidemic rate in the world [1, 2]. Obesity is a state of energy imbalance resulting from excessive food intake and lack of exercise [3, 4] and contributes to the development of metabolic syndrome, diabetes, cardiovascular disease, atherosclerosis, osteoarthritis, and nonalcoholic fatty liver disease [4–6]. Although exercise and dietary control are effective ways of treating obesity, pharmacological treatment is also an important strategy. Currently available therapeutic agents include sibutramine, orlistat, phentermine, and diethylpropion [7]. However, because of the adverse effects of these agents such as abuse, cardiovascular disease, and overstimulation, dietary supplements and herbal products are being recognized as an alternative therapy.

Many types of brown algae are widely eaten in Korea and Japan. Brown algae contain various minerals and dietary fiber and are used as natural health foods. *Ecklonia cava* is a species of brown alga found abundantly in the neritic regions of Korea and Japan [8]. This alga has received attention due to the medicinal effects of its carotenoids, fucoidans, and phlorotannins [9–13], including anti-inflammatory, antioxidative and antidiabetic effects [14, 15]. With regard to its antidiabetic effects, *Ecklonia cava* extract reduces blood glucose levels and increases insulin levels in streptozotocin-induced diabetic mice, a model of type 1 diabetes [14], and the dieckol-rich extract of *Ecklonia cava* improves glucose and lipid metabolism in C57BL/KsJ-db/db (db/db) mice, a model of type 2 diabetes [16]. However, the effects of *Ecklonia cava* on diet-induced obesity have not been specifically examined. In this study, we investigated the effects of *Ecklonia cava* on body weight, fat content, and hyperglycemia in mice fed a high-fat diet (HFD) and sought the antiobesity mechanisms. In addition, we compared the efficacy of *Ecklonia cava* from different areas (produced in Jeju or Gijang, Korea) on our HFD-induced obese mouse model.

## 2. Materials and Methods

### 2.1. Preparation of EtOAc Fraction of EC Crude Extract

The collected samples of *Ecklonia cava *were air-dried on the shade and ground into powder. The powder was extracted repeatedly with MeOH for 3 hours under reflux condition. The crude extract was partitioned between CH_2_Cl_2_ and H_2_O. The organic layer was evaporated and repartitioned between *n*-hexane and 85% aq. MeOH. The aqueous layer was re-partitioned between *n*-BuOH and H_2_O, and then the *n*-BuOH layer was fractionated with EtOAc and H_2_O. The EtOAc fraction was used for HFD-induced obese mouse model experiment.

### 2.2. Determination of Total Polyphenolic Content

The total phenol content was determined using the Folin-Ciocalteu method [17]. An aliquot (20 *μ*L) of each sample or standard solution was mixed with 250 *μ*L of dd H_2_O and 250 *μ*L of Folin-Ciocalteu's phenol reagent. Then, 500 *μ*L of 35% Na_2_CO_3_ solution was added to the mixture followed by incubating at ambient temperature in the dark for 20 min. The absorbance against a blank was measured at 750 nm. The results were expressed as mg tannic acid equivalent (TAE)/g extract (dw).

### 2.3. HPLC Analysis

A portion of EtOAc fraction was subjected to silica gel column chromatography with gradient mixtures of chloroform and methanol. Each of chromatographic fractions was analyzed using an HPLC system (Dionex P580 model) equipped with Varian RI detector and a YMC ODS-A (250 × 4.6 mm) fractionation column with a flow rate of 1 mL/min (eluting solvents, 30% and 40% aq. methanol). Identification and quantification of phlorotannins were carried out by comparing the retention times and the peak areas, respectively, with those of phlorotannin standards. Sample aliquots were filtered through C18 SPE Maxi Clean Cartridge filter prior to injection. The authentic samples of standard phlorotannins (phloroglucinol, triphlorethol A, eckol, eckstolonol, phlorofucofuroeckol A, dieckol, 6,6′-bieckol, 8,8′-bieckol, fucofuroeckol A) were directly isolated from *Ecklonia cava*, and their chemical structures were confirmed by comparing with data reported in the literature.

### 2.4. Animals

C57BL/6 mice were obtained from the Korea Research Institute of Bioscience and Biotechnology (Daejeon, South Korea). Mice were maintained under specific pathogen-free conditions in a temperature-controlled room (23 ± 1°C) in a 12 h light/dark cycle with ad *libitum* access to food and water at the Animal Care Center, Lee Gil Ya Cancer and Diabetes Institute, Gachon University of Medicine and Science, South Korea. All animal experiments were approved by the Institutional Animal Care and Use Committee of the Lee Gil Ya Cancer and Diabetes Institute.

### 2.5. Induction of Obesity and Treatment with EtOAc Fraction of **Ecklonia cava ** Extract

At 6 weeks of age, male mice were provided with either a HFD (60% fat) or normal chow (5.4% fat). After 3 weeks, mice were randomly divided into four groups (*n* = 5–8 in each group): the normal chow group (NC), the phosphate-buffered saline- (PBS-) treated HFD group (PBS-HFD), the Jeju-*Ecklonia cava*- (CA-) treated HFD group (CA-HFD), and the Gijang-*Ecklonia cava*- (G-CA-) treated HFD group (G-CA-HFD). CA or G-CA (200 mg/kg in PBS) was given by oral intubation daily for 8 weeks. The PBS-HFD group was given the same volume of PBS by oral intubation. Body weight and food consumption were measured weekly. At the end of 8 weeks of treatment, animals were killed and tissues were removed for various biochemical measurements.

### 2.6. Measurement of Fat Mass

Fat mass was determined using ^1^H mini-spec system (Bruker, Karlsruhe, Germany) at 8 weeks of treatment. This equipment allowed us to analyze body fat weight without sedating the mice. After 8 weeks of CA and G-CA treatment, fat pads (subcutaneous, epididymal, perirenal, and mesenteric) were collected, and the weights were measured.

### 2.7. Plasma Analysis

After 8 weeks of CA and G-CA treatment, blood samples were collected from the orbital sinus under anesthesia after 3 hours of food deprivation. Blood samples were centrifuged at 3000 g for 20 min, and serum levels of alanine aminotransferase (ALT), aspartate aminotransferase (AST), cholesterol, triglycerides, low-density lipoprotein (LDL)-cholesterol and high-density lipoprotein (HDL)-cholesterol were measured using Beckman Coulter AU 480.

### 2.8. Measurement of Blood Glucose Levels

After 7 weeks of CA and G-CA treatment, mice were not fed for 14 h, and then glucose levels were measured in the tail vein blood with a glucometer.

### 2.9. Intraperitoneal Glucose Tolerance Test

At 7 weeks of treatment, mice were fasted for 14 h and then a glucose solution (2 g/kg body weight in PBS) was administered intraperitoneally. Blood glucose levels were measured at 0, 30, 60, 90, and 120 minutes after glucose injection.

### 2.10. Analysis of mRNA by Quantitative Real-Time PCR

Total RNA was isolated from the adipose and liver tissue, and cDNA was synthesized using a PrimeScript 1st strand cDNA synthesis kit (Takara). Quantitative real-time PCR was performed using the Power SYBR Green Master Mix (Applied-Biosystems) and Applied Biosystem Prism 7900HT sequence detection system. PCR was carried out and stopped at 40 cycles (2 minutes at 50°, 10 minutes at 95°, and 40 cycles of 10 seconds at 95° and 1 minute at 60°). The primer sequences used are shown in Table 1. Relative copy number was calculated using the threshold crossing point (*C*_*t*_) as calculated by the ΔΔ*C*_*t*_ calculations.

### 2.11. Immunoblot Analysis

Samples were prepared from lysates of liver tissue in 50 mM Tris-HCl (pH 7.5), 1% SDS, 150 mM sodium chloride, 10% glycerol, 1 mM EDTA, 1 mM sodium orthovanadate, 1 mM sodium pyrophosphate, 1 mM phenylmethylsulfonyl fluoride, and protease inhibitor cocktail. The protein samples were centrifuged at 10,000 g for 10 min to remove debris and stored at −70°C until use. Protein samples were separated by 10% SDS-polyacrylamide gel electrophoresis. For immunoblots, proteins were electro-transferred to a polyvinylidene fluoride membrane (Schleicher & Schuell), and nonspecific binding was blocked with 2.5% nonfat milk in Tris-buffered saline. The membrane was immunoblotted with anti-AMP-activated protein kinase (AMPK) antibody or anti-phospho-AMPK (Cell Signaling, 1 : 1000). The primary antibodies were detected using horseradish peroxidase-conjugated anti-rabbit IgG (Santa Cruz). Specific binding was detected using the Super Signal West Dura Extended Duration Substrate (Pierce) and exposure to RAS-4000 system (Fuji film). The band density was quantified by the software Multigauge version 3.1 (Fuji film).

### 2.12. Oil Red O Staining

Liver pieces were embedded in optimal cutting temperature compound. Frozen liver sections were cut at 10 *μ*m thickness and stained with Oil Red O and Mayer's hematoxylin solution for microscopy.

### 2.13. Quantification of Liver Triglyceride Content

Liver tissue (50 mg) was homogenized with ethanolic KOH (2 parts EtOH: 1 part 30% KOH) for overnight, and then KOH and distilled water were added to the homogenate. After centrifugation (1000 g, for 5 min), supernatant was transferred into a new microtube and mixed with 1 M MgCl_2_. The sample was incubated for 10 min on ice and then centrifuged at 1000 g for 5 min. Triglycerides content was measured in the upper phase solution using a Cleantech TG-S kit (Asan Pharmaceutical Company).

### 2.14. Statistical Analysis

Data are presented as mean ± SE. The significance of differences was analyzed with the 1-way ANOVA with the Duncan procedure using SPSS ver. 10.0 (SPSS Inc.) The value of statistical significance was set at *P* < 0.05.

## 3. Results

### 3.1. Identification of Phlorotannins in CA and G-CA Extracts

Total polyphenolic contents of the EtOAc fraction of CA and G-CA was 68.78 and 79.70 mg/g, respectively. Phlorotannin composition was determined by HPLC analysis (Table 2). Eckol was abundant polyphenol in both G-CA and CA. Phloroglucinol content in G-CA was higher than that in CA, while triphlorethol A, eckol, eckstolonol, phlorofucofuroeckol A, and dieckol contents in CA were much higher than those in G-CA. 8,8′-bieckol and fucofuroeckol A were found only in G-CA and CA, respectively.

### 3.2. Reduction of Body Weight in G-CA-HFD Mice

**Figure 1:**
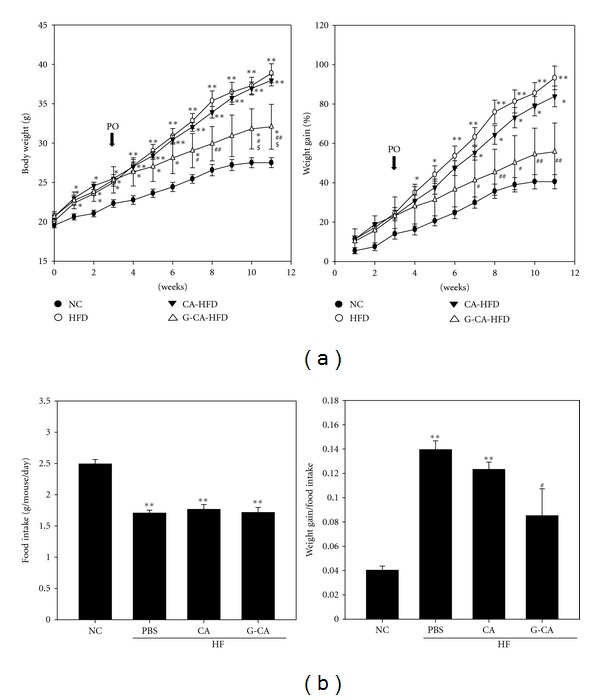
Effect of CA or G-CA extracts on body weight and food intake. Three weeks after beginning a high-fat diet, C57BL6 mice were orally administered CA or G-CA extract (200 mg/kg body weight) or PBS daily for 8 weeks. (a) Body weights were monitored weekly. (b) Food intake was measured weekly. Values are the average weight of food consumed/mouse/day. NC: untreated, normal chow diet, *n* = 7; PBS: PBS-treated, high-fat diet (HFD), *n* = 6; CA: CA-treated, HFD, *n* = 5; and G-CA: G-CA-treated, HFD, *n* = 5. Values are mean ± SE. *P < 0.05, **P < 0.01 versus NC group; ^#^ *P* < 0.05, ^##^ *P* < 0.01 versus PBS-HFD group; ^$^ *P* < 0.05 versus CA-HFD. PO is an abbreviation of per oral.

To examine whether treatment with CA or G-CA affects body weight gain in HFD-induced obese mice, we measured the body weight in CA- or G-CA-treated mice. G-CA-HFD mice had significantly lower body weights and significantly less weight gain (40% decrease) as compared with the PBS-HFD group, whereas the CA-HFD group was not different from the PBS-HFD group (Figure 1(a)). Mice fed a HFD ate significantly less chow than mice fed a normal diet, and the amount of food consumed per day over the 8-week period was not significantly different among the PBS-HFD, CA-HFD, or G-CA-HFD groups (Figure 1(b) left). The food efficiency ratio of the G-CA-HFD mice was significantly lower than that of the PBS-HFD mice (Figure 1(b) right).

### 3.3. Reduction of Adiposity, ALT, and Cholesterol in G-CA-HFD Mice

**Figure 2:**
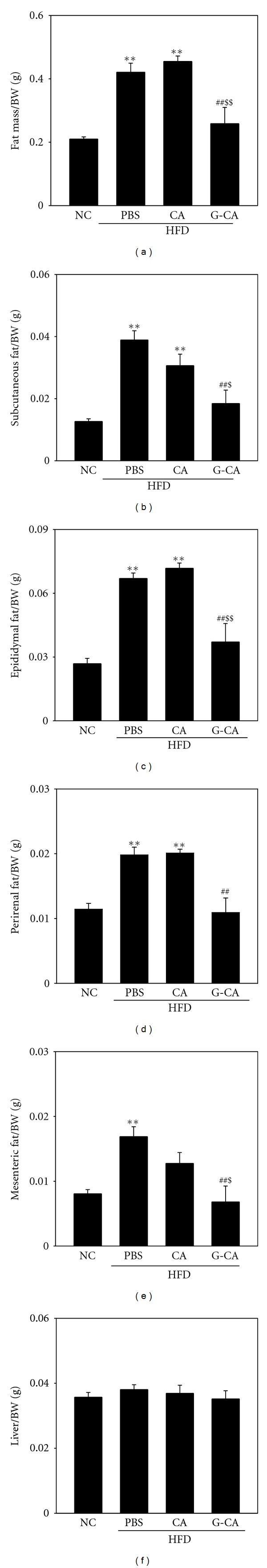
Effect of CA or G-CA extracts on total fat mass and fat tissue weight. Three weeks after beginning a high-fat diet, C57BL6 mice were orally administered CA or G-CA extract (200 mg/kg body weight) or PBS daily. (a) After 8 weeks of CA and G-CA treatment, total fat mass was measured by mini-spec. After 8 weeks of CA and G-CA treatment, ((b)–(e)) fat pads and (f) liver tissue were collected and weighed. Values are the average tissue weight as a proportion of body weight. NC: untreated, normal chow diet; PBS: PBS-treated, high-fat diet (HFD); CA: CA-treated, HFD; G-CA: G-CA-treated, HFD; *n* = 5/group. Values are mean ± SE. **P < 0.01 versus NC group; ^##^ *P* < 0.01 versus PBS-HFD group; ^$^ *P* < 0.05, ^$$^ *P* < 0.01 versus CA-HFD.

Nuclear magnetic resonance measurements were conducted to evaluate body composition. At the end of the treatment, the total body fat content (Figure 2(a)) and the subcutaneous, epididymal, perirenal, and mesenteric fat pad weights (Figures 2(b)–2(e)) of G-CA-HFD mice were significantly reduced as compared with the PBS-HFD group. The liver weights were not different among groups (Figure 2(f)). Plasma analysis showed that ALT, AST, total cholesterol, HDL-cholesterol, and LDL-cholesterol levels were significantly increased in PBS-HFD mice as compared with NC mice. Plasma ALT and cholesterol levels were significantly reduced in G-CA-HFD mice as compared with PBS-HFD mice. G-CA treatment did not affect plasma AST, triglycerides, HDL-cholesterol, or LDL-cholesterol levels compared with PBS-HFD mice (Table 3).

### 3.4. Reduction of Blood Glucose Levels in G-CA-HFD Mice

**Figure 3:**
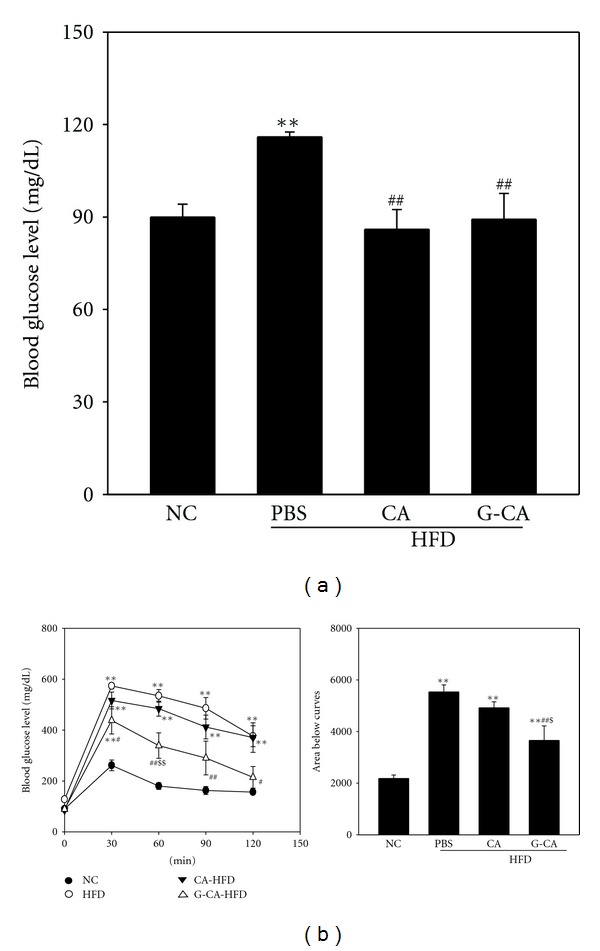
Effect of CA or G-CA extracts on blood glucose levels and glucose tolerance. Three weeks after beginning a high-fat diet, C57BL6 mice were orally administered CA or G-CA extract (200 mg/kg body weight) or PBS daily. At 7 weeks of treatment, (a) blood glucose levels were measured in mice fasted overnight (*n* = 6–8) and (b) intraperitoneal glucose tolerance tests were performed (200 mg/kg glucose body weight, *n* = 5). Values are mean ± SE. NC: untreated, normal chow diet; PBS: PBS-treated, high-fat diet (HFD); CA: CA-treated, HFD; G-CA: G-CA-treated, HFD. Values are mean ± SE. *P < 0.05, **P < 0.01 versus NC group; ^#^ *P* < 0.05, ^##^ *P* < 0.01 versus PBS-HFD group; ^$^ *P* < 0.05, ^$$^ *P* < 0.01 versus CA-HFD.

We measured blood glucose levels, because HFD-induced obese mice are a model for insulin resistance [18]. Plasma blood glucose levels were significantly decreased in CA-HFD and G-CA-HFD mice compared with PBS-HFD mice at 7 weeks of treatment (Figure 3(a)). We also performed intraperitoneal glucose tolerance tests at 7 weeks. Blood glucose levels in the G-CA-HFD group were significantly lower at all time points following glucose injection compared with the PBS-HFD group (Figure 3(b), left). Area under the curve of the G-CA-HFD group was decreased 25.2% compared with the PBS-HFD group (Figure 3(b), right).

### 3.5. Increase in mRNA Expression of Adipogenesis-Related Genes in Adipose Tissue of G-CA-HFD Mice

**Figure 4:**
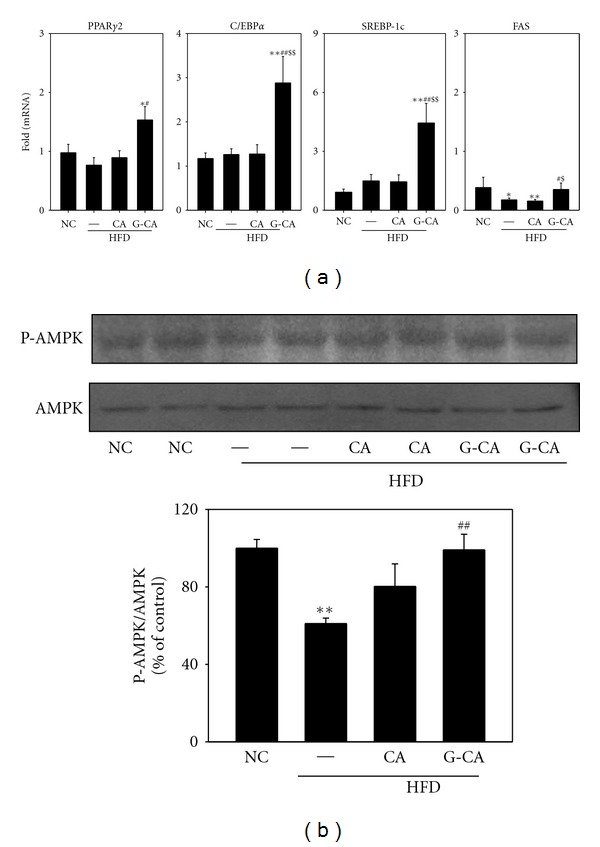
Effect of CA or G-CA extracts on adipogenic gene expression and AMPK phosphorylation. Three weeks after beginning a high-fat diet, C57BL6 mice were orally administered CA or G-CA extract (200 mg/kg body weight) or PBS daily for 8 weeks. (a) PPAR*γ*2, C/EBP*α*, SREBP-1c, and Fas mRNA levels were measured in epididymal fat pads by quantitative real-time PCR. Values are expressed as fold change compared with the NC group. (b) Phospho (P)-AMPK and AMPK protein expression in epididymal fat pads was analyzed by western blot (upper panel) and quantified (lower panel). Values are mean ± SE. NC: untreated, normal chow diet; PBS: PBS-treated, high-fat diet (HFD); CA: CA-treated, HFD; G-CA: G-CA-treated, HFD. *P < 0.05, **P < 0.01 versus NC group; ^#^ *P* < 0.05, ^##^ *P* < 0.01 versus PBS-HFD group; ^$^ *P* < 0.05, ^$$^ *P* < 0.01 versus CA-HFD.

In order to investigate whether CA or G-CA treatment changes adipogenesis-related gene expression, we analyzed the expression of peroxisome proliferator-activated receptor (PPAR)*γ*2, CCAAT-enhancer-binding protein (C/EBP)*α*, sterol regulatory element-binding protein (SREBP)-1c, and FAS mRNA in epididymal adipose tissue after 8 weeks of CA and G-CA treatment. mRNA levels for the adipogenic related transcription factors, PPAR*γ*2, C/EBP*α*, and SREBP-1c, were not changed by HFD. The mRNA expression of these genes was significantly increased in G-CA-HFD mice compared with PBS-HFD mice, but not changed in CA-HFD mice (Figure 4(a)). FAS mRNA levels were significantly decreased in PBS-HFD mice and CA-HFD mice as compared with the NC group and the G-CA-HFD group had significantly higher FAS mRNA levels as compared with PBS-HFD mice and CA-HDF mice (Figure 4(a)). Next we performed western blots to determine whether CA or G-CA treatment altered the phosphorylated protein levels of AMPK, which is involved in fatty acid oxidation. Phosphorylated AMPK levels of mice fed a HFD were decreased as compared with the NC group and whereas those of G-CA-HFD mice group were significantly increased (Figure 4(b)).

### 3.6. Decrease in mRNA Expression of Inflammatory Genes in G-CA-HFD Mice

**Figure 5:**
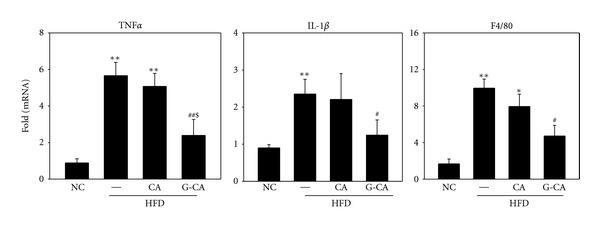
Effect of CA and G-CA extracts on inflammatory gene expression. Three weeks after beginning a high-fat diet, C57BL6 mice were orally administered CA or G-CA extract (200 mg/kg body weight) or PBS daily for 8 weeks. TNF*α*, IL-1*β*, and F4/80 mRNA levels were measured in epididymal fat pads. Values are expressed as fold change compared with the NC group. Values are mean ± SE. NC: untreated, normal chow diet; PBS: PBS-treated, high-fat diet (HFD); CA: CA-treated, HFD; and G-CA: G-CA-treated, HFD. *P < 0.05, **P < 0.01 versus NC group; ^#^ *P* < 0.05, ^##^ *P* < 0.01 versus PBS-HFD group; ^$^ *P* < 0.05 versus CA-HFD.

Because obesity and type 2 diabetes are accompanied by chronic inflammation, inflammatory cytokine levels are increased in HFD-induced obese mice [19]. Therefore, we analyzed whether CA or G-CA treatment can attenuate inflammatory signaling in the epididymal adipose tissue of HFD-induced obese mice. mRNA levels of the cytokines, tumor necrosis factor (TNF)-*α*, interleukin (IL)-1*β* and the macrophage marker F4/80 were significantly increased in PBS-HFD mice as compared with NC mice. G-CA-HFD mice had significantly reduced TNF-*α*, IL-1*β*, and F4/80 mRNA levels compared with PBS-HFD mice (Figure 5).

### 3.7. Reduced Intrahepatic Lipid Accumulation and Hepatic Lipogenic Gene mRNA Expression in G-CA-HFD Mice

**Figure 6:**
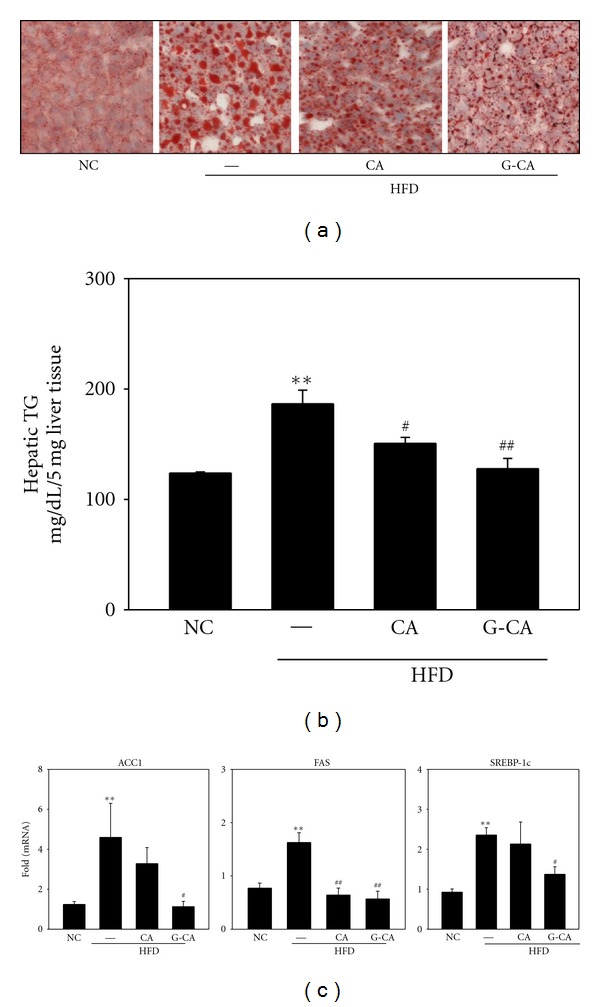
Effects of CA and G-CA extracts on hepatic steatosis. Three weeks after beginning a high-fat diet, C57BL6 mice were orally administered CA or G-CA extract (200 mg/kg body weight) or PBS daily for 8 weeks. (a) Oil red O staining was performed on frozen liver sections. (b) Hepatic triglyceride (TG) content was measured in liver tissue. (c) ACC1, Fas, and SREBP1c mRNA levels were analyzed in liver tissue. Values are expressed as fold change compared with the NC group. Values are mean ± SE. NC: untreated, normal chow diet; PBS: PBS-treated, high-fat diet (HFD); CA: CA-treated, HFD; and G-CA: G-CA-treated, HFD. **P < 0.01 compared with NC group. ^#^ *P* < 0.05, ^##^ *P* < 0.01 compared with PBS-HFD group.

Because the elevation of hepatic lipid during a HFD causes nonalcoholic fatty liver [20], we assessed the lipid content in frozen sections of liver. Lipid droplet accumulation was obviously increased in PBS-HFD mice, whereas lipid droplets were reduced in CA-HFD and G-CA-HFD mice compared with PBS-HFD (Figure 6(a)). Hepatic triglyceride levels were significantly increased in PBS-HFD mice as compared with NC mice. However, the hepatic triglyceride content in CA-HFD and G-CA-HFD mice was significantly reduced as compared with PBS-HFD mice, 19% and 32%, respectively (Figure 6(b)). When we examined the mRNA expression of genes that control lipid metabolism in the liver, we found that ACC1, FAS, and SREBP-1c mRNA levels were significantly increased in PBS-HFD mice as compared with NC mice. The expression of these genes was significantly decreased in G-CA-HFD mice as compared with PBS-HFD mice, and FAS levels were also significantly decreased in CA-HFD mice (Figure 6(c)).

## 4. Discussion

 In the present study, we investigated the antiobesity and glucose-lowering effects of extracts from the brown alga, *Ecklonia cava* in HFD-induced obese mice and compared the activity of algae from different areas: Jeju (CA) and Gijang (G-CA). After 8 weeks of treatment, we observed a significant antiobesity effect of G-CA extract in HFD-induced obese mice, evidenced by decrease of body weight gain relative to PBS-HFD mice, without any change in food intake. Total fat mass and peripheral fat fad weight were also decreased in G-CA-treated mice. In contrast, CA treatment did not result in significant reductions in either body weight or fat mass, suggesting that G-CA has a more potent antiobesity effect than CA. Both extracts significantly reduced fasting blood glucose; however G-CA had a more potent glucose-lowering effect than CA as determined by glucose tolerance tests.

 The adipose tissue plays an important role in whole-body energy homeostasis, and thus, its functional disorder has relevance for metabolic syndrome and diabetes. The transcription factors PPAR*γ* and C/EBP*α* are key regulators of adipocyte differentiation and promotion of lipid storage. SREBP1 regulates lipogenesis-related gene expression such as FAS and activates PPAR*γ* gene expression [21]. In our study, the mRNA expression of adipogenesis-related genes such as PPAR*γ*, C/EBP*α*, SREBP-1c and FAS did not increase, but rather slightly decreased in epididymal fat pads as a result of the HFD. This may be part of an adaptive response to limit further fat deposition during a prolonged HFD [22], and others have also found that PPAR*γ*, C/EBP*α* and SREBP mRNA levels were decreased in the adipose tissue of a different obese mouse model [23, 24]. Furthermore, mRNA levels of SREBP-1c in obese patients were lower than those in normal weight subjects, but mRNA levels increased along with weight loss [25, 26]. The reduction of SREBP-1c expression has relevance to lowered action or concentration of insulin, modifiable along with weight reduction [25]. We found that G-CA treatment increased the mRNA expression levels of PPAR*γ*, C/EBP*α*, SREBP-1c, and FAS in epididymal fat pads of HFD-fed mice. As well, G-CA treatment increased the phosphorylation of AMPK, a key regulator of fatty acid oxidation. These results suggest that G-CA treatment improved intracellular fatty acid metabolism by improving adipogenic gene expression and fatty acid oxidation.

 Inflammation and macrophage infiltration in adipose tissue is associated with obesity and insulin resistance [27–29]. Cytokines produced from infiltrated macrophages and adipocytes regulate adipose tissue metabolism [30]. The inflammatory cytokine, TNF-*α*, decreases SREBP1 expression levels in adipose tissue, thus disturbing adipogenic gene expression and adipogenesis regulation [31]. As cytokines released from adipose tissue lead to insulin resistance and *β*-cell failure [32, 33], increase of TNF-*α* and IL-1*β* expression is important in the pathogenesis of type 2 diabetes. We found that mRNA expression levels of TNF-*α*, IL-1*β* and the macrophage maker, F4/80, were increased in HFD-fed mice but were decreased in G-CA-treated mice. The anti-inflammatory effect of G-CA may contribute to the reduction of obesity and improvement of glucose tolerance.

 Nonalcoholic fatty liver disease is associated with metabolic syndrome. Excessive triglyceride accumulation in hepatocytes changes lipid metabolism in the liver [34]. In our study, the HFD increased fat and triglyceride accumulation and lipogenic gene expression; however, both CA and G-CA treatment lowered these measures, with G-CA being more effective than CA. Serum ALT levels, a marker of liver injury, were also significantly increased in HFD-fed mice, but decreased in G-CA-treated mice. These results indicate that G-CA and CA have an ameliorating effect on fatty liver through the impaired hepatic dyslipidemia and G-CA is more effective than CA.

*Ecklonia cava* has beneficial bioactive components including phlorotannins and polysaccharides such as alginic acid, fucoidans, pyropheophytin, tripeptides, and oxylipin. Of these, phlorotannins including phloroglucinol, phloroglucinol tetramer, eckol, phlorofucofuroeckol A, dieckol, 8,8′-bieckol, and dioxinodehydroeckol have been studied for their pharmacological activity. In our study, G-CA was consistently more effective than CA in reducing obesity, glucose levels, and related biochemical parameters in HFD-fed mice. The content of active components in some plants depends on environmental conditions, weather, geographic location, and soil conditions. According to our HPLC analysis, CA and G-CA contain phloroglucinol, eckol, phlorofucofuroeckol A, and dieckol in common as major components. However, triphlorethol A and eckstolonol were found as major components in CA while they were minor components in G-CA. In addition, 8,8′-bieckol was found as major components in G-CA. Total polyphenol concentration of G-CA was higher than that of CA. Whether these differences in content and kinds of active components contribute to different pharmacological effects between CA and G-CA remains to be tested in the future.

## 5. Conclusion

This study showed that G-CA improved obesity and glucose levels by anti-inflammatory actions and improvement of lipid metabolism. Therefore, G-CA have the potential to be developed as functional food and antiobesity therapeutic agent.

## Figures and Tables

**Table 1:** Primer sequences of mouse mRNA.

Target	Forward primer	Reverse primer
PPAR*γ*2	CACCAGTGTGAATTACAGCAAATC	ACAGGAGAATCTCCCAGAGTTTC
C/EBP*α*	GCGCAAGAGCCGAGATAAAG	CGGTCATTGTCACTGGTCAACT
SREBP1c	GGAGCCATGGATTGCACATT	GGCCCGGGAAGTCACTGT
FAS	GCTGCGGAAACTTCAGGAAAT	AGAGACGTGTCACTCCTGGACTT
TNF*α*	CCAACGGCATGGATCTCAAAGACA	AGATAGCAAATCGGCTGACGGTGT
IL-1*β*	CTACAGGCTCCGAGATGAACAAC	TCCATTGAGGTGGAGAGCTTTC
F4/80	TCATCAGCCATGTGGGTACAG	CACAGCAGGAAGGTGGCTATG
ACC1	ACGCTCAGGTCACCAAAAAGAAT	GTAGGGTCCCGGCCACAT

**Table 2:** Polyphenol content in CA and G-CA extracts as determined by HPLC.

Polyphenolic component	CA	G-CA
	mg/g extract	mg/g extract
Phloroglucinol	0.80	3.17
Triphlorethol A	1.29	0.23
Eckol	12.98	4.72
Eckstolonol	12.78	0.13
Phlorofucofuroeckol A	11.04	2.57
Dieckol	16.56	1.12
6,6^′^-Bieckol	1.01	0.83
8,8^′^-Bieckol	0.00	1.79
Fucofuroeckol A	1.21	0.00
Others	42.33	85.44

**Table 3:** Plasma biochemical parameters.

	NC	PBS-HFD	CA-HFD	G-CA-HFD
ALT (U/L)	41.97 ± 1.20	60.25 ± 3.00^∗∗^	46.83 ± 4.76^#^	32.45 ± 4.92^## $^
AST (U/L)	77.77 ± 20.26	147.23 ± 14.73^∗^	151.13 ± 17.25^∗^	138.48 ± 20.24^∗^
Cholesterol (mg/dL)	64.63 ± 3.98	174.45 ± 14.07^∗∗^	163.25 ± 8.16^∗∗^	125.90 ± 5.86^∗∗##$^
Triglycerides (mg/dL)	61.13 ± 5.90	76.18 ± 5.27	87.85 ± 14.36	66.03 ± 9.09
HDL-cholesterol (mg/dL)	108.90 ± 4.38	203.93 ± 11.56^∗∗^	189.28 ± 3.42^∗∗^	175.98 ± 11.39^∗∗^
LDL-cholesterol (mg/dL)	33.23 ± 1.85	54.75 ± 4.31^∗^	58.58 ± 9.38^∗^	45.05 ± 3.82

NC: untreated, normal chow diet, PBS-HFD: PBS-treated, high-fat diet, CA-HFD: CA-treated, high-fat diet, and G-CA-HFD: G-CA-treated, high-fat diet. ^∗^ *P* < 0.05, ^∗∗^ *P* < 0.01 versus NC group; ^#^ *P* < 0.05, ^##^ *P* < 0.01 versus PBS-HFD group; ^$^ *P* < 0.05 versus CA-HFD.
